# Prevalence of ketosis in dairy cows in milk shed areas of Odisha state, India

**DOI:** 10.14202/vetworld.2016.1242-1247

**Published:** 2016-11-14

**Authors:** Sangram Biswal, Dhruba Charan Nayak, Kautuk Kumar Sardar

**Affiliations:** 1 Department of Veterinary Medicine, College of Veterinary Science and Animal Husbandry, Orissa University of Agriculture and Technology, Bhubaneswar, Odisha, India; 2 Department of Pharmacology and Toxicology, College of Veterinary Science and Animal Husbandry, Orissa University of Agriculture and Technology, Bhubaneswar, Odisha, India

**Keywords:** age, breed, dairy, ketosis, lactation, milk cows, prevalence

## Abstract

**Aim:**

The present study was conducted to ascertain the prevalence of ketosis in dairy cows in dairy herds, milksheds, and mixed population of milk cows selected randomly in milkshed areas of Odisha state, India.

**Materials and Methods:**

The investigation was conducted in 280 private dairy herds with variable herd size of 10-15 cows comprising crossbred Jersey cows (CBJ), crossbred Holstein Friesian (CHF) cows, and indigenous local breeds. The analysis of urine (Rothera’s test), milk (Ross test), and blood samples of 2760 test cows were conducted through qualitative assessment by Rothera’s test and Ross test, respectively, for the presence of ketone bodies to screen the ketotic animals. Cut-points have been decided based on β-hydroxybutyric acid level (≥1.2-1.4 mmol/L) in milk.

**Results:**

We noted positive cases of ketosis with a prevalence rate of 36.7% (1014/2760) entailing 27.2% in clinical ketosis and 9.6% in subclinical ketosis. The breed wise incident rate was recorded to be the highest (38.0%) in CBJs. The age-wise prevalence rate was found to be the highest (40.8%) in the age group of 5.5-6.5 years. The season wise prevalence rate in 5^th^ calver was recorded to be the highest (38.6%) in summer season as compared to other seasons. The prevalence of ketosis was observed to be the highest at 56.7% on the first stage of lactation at the 1^st^ month after 2 weeks. The incidence rates for clinical and subclinical ketosis were found to be 25.2%; 12.2%, 26.6%; 11.2% and 30.3%; 2.9% in CBJ, CHF and indigenous cows, respectively. The breed wise overall prevalence rate was recorded to be 38.0% in CBJ, 37.8% in CHF, and 33.2% in indigenous cows.

**Conclusion:**

Ketosis and subclinical ketosis is highly prevalent metabolic disorder and has severe effect on the production status of affected animal and needs to be prevented, rather than treated, by maintaining cows in good and healthy conditions. We have attempted to give great attention for diagnosis, management, and control of this disease during risk stage to prevent economic loss sustained by the dairy farmers of Eastern India.

## Introduction

Ketosis is a production disease with high intensity of prolonged morbidity causing substantial loss in dairy industry [1,2]. Ketosis has become a very common metabolic disorder in modern dairy production by causing decrease in milk production and increase in prevalence and duration of fresh cow diseases, enhancing time to conception, and augmenting risk of culling. Numerous global studies have indicated that ketosis increases from a low prevalence at the first lactation to a peak level in the fourth lactation with great variation of cumulative lactational prevalence among dairy herds, averaging about 40.0% which can be as high as 80.0% in some herds [3-7].

Ketosis, a multi-factorial disorder of energy metabolism, leads to hypoglycemia and hyperketonemia. The prevalence rate is the highest during the period commencing at calving and extending until about peak lactation [2]. Production diseases are mainly man made problems, which occupy the most key place among the diseases of dairy animals as it directly or indirectly affects the economy of dairy farm and ultimately dairy farmers suffer from huge financial losses due to drastic decrease in milk production [8]. The clinical signs of ketosis often remain ill-understood by the farmers as well as the veterinarians, as a result, the true prevalence of ketosis remains underdiagnosed in the field condition. The prevalence of ketosis in most of the common management systems after calving has not been explored entailing high production loss to the dairy farmers [9].

Ketosis can be diagnosed by measuring ketone bodies present in urine, milk, and blood.

Because of the economic consequences, it is imperative to diagnose ketosis in dairy cows, particularly during early lactation for treatment in advance and prevention of further losses. In this study, attempts have been made to ascertain the frequency of different forms of ketosis in respect of prevalence, age, breed, season, stage of calving and stage of location among the dairy cows in milk-shed areas of Odisha, India. The proposed findings will be a step forward for landless, marginal and progressive dairy owners in India in uplifting their economic output through effective and compliant therapeutic intervention.

## Materials and Methods

### Ethical approval

The experiment was done in accordance with the guidelines provided by the institutional ethical committee and also complies with the country’s laws.

### Place of study

This study was conducted in the Department of Veterinary Medicine, College of Veterinary Science and Animal Husbandry, Orissa University of Agriculture & Technology, Bhubaneswar, Odisha State, India.

### Sample collection and clinical examination

The dairies of milk shed areas under Fisheries and Animal Husbandry Department, Government of Odisha/Odisha State Cooperative Milk Producers’ Federation Limited were selected. There exists a dearth of information on prevalence rates considering the diversity of milk cows in milk shed areas of Odisha, India. The dairies were contacted by the Block Veterinary Officers, Dairy cooperatives, Para-veterinarians, etc., over telephone with regard to prevalence of ketosis in the milk shed areas under the study. The urine, milk, blood samples, in this study, were collected from dairy herds, milk-sheds and mixed population of milk cows selected randomly using simple lottery method as per Snedecor and Cochran (1994). The survey was conducted in 280 private dairy herds with variable herd size of 10-15 cows comprising crossbred Jersey cows (CBJ), crossbred Holstein Friesian (CHF) cows, and indigenous local breeds of cows. The investigation on the prevalence of ketosis was based on the clinical and subclinical cases screened through the Rothera’s test for urine and Ross test for milk for detection of ketone bodies. During the study, 2760 milk-cows of mixed population were selected randomly from the herds in the age group of about 2.5-7.5 years. After obtaining complete history of milk yield, stage of lactation, age from the dairy owners, the analysis of urine, milk and blood samples of 2760 test-cows conducted through qualitative assessment by Rothera’s test and Ross test, respectively, for the presence of ketone bodies were confirmed for primary ketosis. The baseline survey was conducted to assess the severity of ketosis and acquaintance with dairy farmers followed by collection of samples in second and subsequent visits on different dates, but the cows were sampled once only. The clinical symptoms such as reduced milk yield, inappetance, excess salivation, stomach pain, and acetone (pear drop) smell of milk with rapid breathing were observed in milk cows during sampling, and treated accordingly.

### Examination of urine by Rothera’s test

Urine samples were collected in clean dry glass receptacles in the morning and were subjected to Rothera’s test for detection of ketone bodies at the door-steps of the dairy owners. Briefly describing, 3 ml of urine was taken in a clean dry test tube and was saturated with ammonium sulfate granules. An equal volume (3 ml) of freshly prepared 5% sodium nitroprusside solution was mixed gently in the saturated solution by tilting the test tube. About 1 ml of concentrated ammonium hydroxide solution was layered and allowed to stand for 0.5 min. Development of permanganate like color (purple color) indicated the presence of ketone bodies. The intensity of color index was graded in the following manner for presence of ketone bodies: No color index of purple color: −ve; Faintly purple color: ±ve; Slightly purple color: +ve; Purple color: ++; Deep purple color: +++; Very deep purple color (Black): ++++.

### Examination of milk by Ross test

Milk samples collected from each lactating cow randomly in clean and dry test tubes in the morning were subjected to Ross test. Milk sample (5 ml) was saturated with ammonium sulfate and 1-2 drops of freshly prepared 5% sodium nitroprusside solution were added to it followed by addition of a flake of sodium hydroxide into the test tube. The solution was shaken gently and slowly and allowed to stand for 5 min. The development of purple color was graded. The gradation of color index was taken for assessment of subclinical cases with slow reduction of milk yield. The intensity of color indices with the gradation of +, ++, +++, and ++++ along with clinical manifestations observed were the criteria to assess the clinical forms of subclinical and clinical ketosis. Moreover, to screen the cows for ketosis at cow-side, the milk was tested semiquantitatively to determine the β-hydroxybutyric acid (BHBA) level (≥1.2-1.4 mmol/L) in milk.

### Statistical analysis

Date recorded, in this study, were analyzed statistically applying independent Student’s t-test using SPSS version 17.0. The significance difference (p<0.05) with respect to different seasons to assess the seasonal prevalence of ketosis in relation to stage of calving. Single-variate statistical method was followed in the present study, and prevalence percentage of ketosis has been studied with respect to different covariates. The dairy cows (n=24) selected from the similar environmental conditions, feeding and managemental practices were treated as control group to establish the association. From each parity and each breed, one clinically healthy milk cow was selected which would have come to a total of 90 baseline cows. However, the control group was reduced to 24 only due to noncooperation of dairy farmers in some villages under study.

## Results

The analysis of ketone bodies in urine through Rothera’s test and Ross test for milk conducted in the Department of Veterinary Medicine, College of Veterinary Science and Animal Husbandry revealed that 1014 of 2760 lactating cows were found positive for ketosis with an overall prevalence rate of 36.7% (27.2% for clinical cases and 9.6% for subclinical cases). The prevalence rates for clinical and subclinical ketosis were found to be 25.2%. 12.2%; 26.6%; 11.2% and 30.3%; 2.9% in CBJ, CHF and indigenous (Ind.) cows, respectively (Table-1).

**Table 1:** Prevalence of ketosis in relation to age, breed, stage of lactation in lactating cows (n=1014).

Parameters	Age-wise					Number of cows found ketotic	Stage of lactation	
	
	2.5-3.5 years 1^st^ calver	3.5-4.5 years 2^nd^ calver	4.5-5.5 years 3^rd^ calver	5.5-6.5 years 4^th^ calver	6.5-7.5 years 5^th^ calver		Clinically ketotic	Subclinically ketotic
Number of cases (CBJ)	66 (206)	94 (272)	64 (170)	154 (364)	94 (230)	472 (1242)	321	151
Percentage	32.0	34.6	37.6	42.3	40.9	38.0	25.2	12.2
Number of cases (CHF)	68 (218)	16 (48)	52 (140)	101 (238)	78 (190)	315 (834)	222	93
Percentage	31.2	33.3	37.1	42.4	41.1	37.8	26.6	11.2
Number of cases (Ind.)	57 (184)	11 (35)	30 (93)	63 (178)	66 (194)	227 (684)	207	20
Percentage	30.9	31.4	32.3	35.4	34.0	33.2	30.3	2.9

Values in parentheses denote number of cows examined. CBJ=Crossbred Jersey cows, CHF=Crossbred Holstein Friesian cows, Ind.=Indigenous cows

The breed wise screening revealed an overall prevalence of 36.7% (1014/2760), out of which the clinically ketotic and subclinically ketotic animals were recorded as 27.2% and 9.6%, respectively. The breed wise overall prevalence % was recorded to be highest in CBJ, followed by CHF and indigenous cows.

The frequency of age wise prevalence (40.8%) among the lactating cows in the age group of 5.5-6.5 years (4^th^ calver) was followed by 6.5-7.5 years (5^th^ calver), 4.5-5.5 years (3^rd^ calver), 3.5-4.5 years (2^nd^ calver), and 2.5-3.5 years (1^st^ calver). The breed wise prevalence under study prevailed the highest (42.4%) in 4^th^ calver in CHF cows which was trailed by 42.3% in 4^th^ calver in CBJ cows and 35.4% in 4^th^ calver in indigenous cows (Table-1).

The seasonal prevalence of ketosis was significantly (p<0.05) higher in summer with an overall prevalence of 38.6% and the prevalence in autumn season was the highest (40.29%) in 4^th^ calver with an overall prevalence of 36.1% (Table-2). Similarly, the frequency of ketosis in dew season was found to be 39.5% in 4^th^ calver with an overall prevalence of 34.9%. The prevalence of ketosis in winter season was recorded as the highest (40.9%) in 4^th^ with an overall prevalence rate of 37.3%. The prevalence in spring season was maximum (40.0%) in the 4^th^ calver with an overall prevalence of 34.9% (Table-2). The seasonal prevalence of ketosis was recorded to be the highest in summer followed by rainy, winter, autumn, dew, and spring season.

**Table 2:** Seasonal prevalence of ketosis in relation to the stage of calving in lactating cows (n=1014)

Season	1^st^ calver		2^nd^ calver		3^rd^ calver		4^th^ calver		5^th^ calver		Total number of cows	
					
	Number of cows examined	Number of cows found ketotic (%)	Number of cows examined	Number of cows found ketotic (%)	Number of cows examined	Number of cows found ketotic (%)	Number of cows examined	Number of cows found ketotic (%)	Number of cows examined	Number of cows found ketotic (%)	Number of cows examined	Number of cows found ketotic (%)
Summer	110	38 (34.5)	62	23 (37.1)	70	27 (38.6)	136	57 (41.9)	109	43 (39.4)	487	188 (38.6)
Rainy	94	32 (34.0)	65	23 (35.4)	68	26 (38.2)	144	60 (41.7)	99	39 (39.4)	470	180 (38.3)
Autumn	104	32 (30.8)	56	18 (32.1)	66	23 (34.8)	134	54 (40.3)	105	41 (39.0)	465	168 (36.1)
Dew	90	26 (28.8)	51	16 (31.8)	67	23 (35.7)	124	49 (39.5)	103	38 (36.8)	435	152 (34.9)
Winter	110	35 (31.8)	60	22 (36.6)	65	24 (36.9)	122	50 (40.9)	102	40 (39.2)	459	171 (37.3)
Spring	100	28 (28.0)	61	19 (31.1)	67	23 (34.3)	120	48 (40.0)	96	37 (38.5)	444	155 (34.9)
Total	608	191 (31.4)	355	121 (34.1)	403	146 (36.2)	780	318 (40.7)	614	238 (38.7)	2760	1014 (36.7)
Mean±SE	101.3±3.7	31.8±1.8^b^	59.2±2.0	20.2±1.1^a^	67.1±0.7	24.3±0.7^b^	130.0±3.8	40.7±1.9^c^	102.3±1.8	39.7±0.8^c^	460.0±7.7	169.0±5.4

Values in parentheses denote percentage; Values are expressed as mean±SE; Mean values with different superscripts (a, b and c) in a column differ significantly at p<0.05. SE: Standard deviation

The prevalence of ketosis in relation to the stages of lactation was recorded as 56.8% in the 1^st^ month, 29.7% in the 2^nd^ month, 7.2% in the 3^rd^ month, 3.6% in the 4^th^ month, 1.3% in the 5^th^ month, 0.8% in the 6^th^ month, 0.4% in the 7^th^ month, and 0.2% in the 8^th^ month of lactation in CBJ indicating the highest percentage of the total prevalence (86.4%) in the first 2 months of lactation. Similarly, the prevalence of ketosis was found to be 53.9% and 60.4% in CHF and indigenous cows in the 1^st^ month of lactation, respectively. The highest overall prevalence of ketosis was recorded to be 87.1% with a range of 84.7-91.6% within 60 days of lactation and the lowest overall prevalence rate of 0.1% was recorded at the 8^th^ month of lactation indicating a rarely occurrence of this metabolic disease at this stage when the milk yield was found to have been substantially reduced for preparing the body for next parturition.

## Discussion

The present investigation on bovine ketosis succeeded for the first time in the milkshed areas of Odisha to record the prevalence of ketosis (36.7%). The prevalence of clinical ketosis and subclinical ketosis 27.2% and 9.6%, respectively, are in agreement with a commensurable prevalence of 36.0% among the CBJ cows in the same locality [10]. In close vicinity of Bhubaneswar city, Odisha, previous workers have also found the prevalence of 38.0% and 40.0% [11,12]. Our findings of the prevalence of 27.2% for clinical ketosis and 9.6% for subclinical ketosis are in agreement with the findings of earlier workers who recorded prevalence of 28.8% for clinical ketosis and 7.2% for subclinical ketosis [10]. Panda (2003) also recorded the prevalence to be 28.8% for clinical ketosis and 21.2% for subclinical ketosis in Odisha [12].

A varied range of prevalence (0-40%) was documented as per the present finding of 36.7% [13]. On the contrary, a wide range of prevalence varying from 2.9% to 59.7% was also recorded [14-17]. Such type of wide variation in prevalence might be attributed to agroclimatic condition, climate change, breed susceptibility, stage of calving, feeding habit, production potential, and managemental practices followed by the dairy owners.

The present finding of the prevalence of subclinical ketosis was in conformity with some prior findings who reported that CBJ cows were found to be more susceptible for subclinical ketosis than CHF cows [18,19]. The prevalence rates, in this study, revealed that the lactating cows from the age group of 2.5-3.5 years to 5.5-6.5 years followed the ascending pattern of prevalence rate with advancement of time up to 6.5 years (i.e., 4^th^ calving stage) [20].

The present investigation revealed the influence of breed susceptibility on prevalence and was manifested as 38.0% in CBJ followed by 37.8% in CHF and 33.1% in indigenous cows which corroborates the pattern of prevalence rates in CBJ (44.8%), CHF (41.5%) and nondescript breed (13.3%) of cows in West Bengal [19]. Our findings of the prevalence rate of 25.2% in CBJ, 26.6% in CHF and 30.3% in indigenous cows for clinical ketosis presenting a mixed pattern of prevalence rate did not support the breed susceptibility in clinical ketosis. However, this finding of the prevalence of 12.2% in CBJ, 11.2% in CHF and 2.9% in indigenous cows for subclinical ketosis demonstrated the highest prevalence rate of subclinical ketosis in CBJ followed by CHF and indigenous cows [18,20].

This pattern of occurrence of ketosis might have cropped up due to the fact that in the prime stage of productive life, the young cows were accomplished with increased body activities and physiological tolerance against adverse conditions and had the greater power of adjustment between input and output physiologically. In advancement of aging process, different physiological processes became gradually slower. But within the age group of 6.5-7.5 years, the prevalence was found to be lower as against the age group of 5.5-6.5 years in every crossbred cow under investigation. This type of phenomenon might be coined with the decreased productivity and low output of glucose and other metabolites.

The ascending pattern of prevalence from lower age group of 3-4 years to higher age group of 5-6 years observed in the present investigation are in agreement with the similar pattern of prevalence in the cows of different age groups [11,20]. This finding of prevalence was the highest (40.76%) in the cows within the age group of 5.5-6.5 years (4^th^ calver) as against the highest prevalence of 44.6% among the cows within the age group of 5-6 years [11]. Contrary to our findings, the higher prevalence of 50.0% and 37.0% were recorded in the cows within the age group of 5-7 and 8-9 years, respectively [15,19,21]. The prevalence rate of ketosis (24.7%) among the milk cows between 3 and 6 years of age was lower than the present findings [17].

The prevalence increased gradually with the increased calving status up to 4^th^ calving period and then declined from 5^th^ calving status [11]. It was also observed that more number of milk cows between the 2^nd^ and 5^th^ calving stage were found to be ketotic as compared to 1^st^ calving stage. It might be elucidated that the cows in very first state of calving were not accomplished with the highest lactation potential as the udder development was still to attain its fulfillment with required amount of secretary tissues for synthesis of milk. The present findings corroborate with earlier findings which reported that ketosis was more prevalent among the multipara milk cows [14,20,22].

The highest prevalence in summer season followed by rainy season and winter season was due mainly to seasonal stress factors. The highest prevalence in summer season in the present study might be attributed to extreme stress and strain due to scorching heat of summer and nonavailability of the free grazing pasture leading to malnutrition [23]. So also in rainy and winter season, the adverse factors arose from the inclement weathers due to incessant rain, chilly atmosphere, and stormy wind flows. Besides, in summer, the lactating cows were maintained mostly with hay and concentrates low in protein [24]. Due to nonavailability of forages or green grasses, the complete diets or mixed rations in proportion of 55.0% of concentrate and 45.0% of forage or green grasses might not be available to the milk cows leading to reduction in feed intake [25]. As detailed above, these factors in adverse conditions might guide the high yielding cows to ketonemia, as a need for increased milk yield increased tissue metabolism to meet the demand for energy homoeostasis leading to excess generation of the ketone bodies [26,27].

Our investigation was confined to the small and marginal dairy farmers in the milk shed areas of Odisha who were deprived of sophisticated managemental system. The milk cows in the milk shed areas were managed in natural village condition on feeding inadequate quantity of rice bran, wheat bran, broken pulses, oil cakes, common salt, mineral mixtures, paddy straw, and the cow-sheds under study were of thatched roofs with kutcha floors with poor sanitary conditions. To satisfy the requirements of milk production, the cow can draw on two sources of nutrients namely, feed intake, and body reserves. During early lactation, the energy intake is insufficient to meet the energy output in milk and the animal is in a negative energy balance. In conventional farming, this is considered to be a normal metabolic situation in high-yielding dairy cows. Cows in early lactation are, therefore, in a vulnerable situation, and any stress that causes a reduction in feed intake may stand more chances for clinical ketosis. The high prevalence of bovine ketosis in winter season in Northern climatic conditions was recorded which exerted the adverse health impacts on dairy cattle [28]. In such areas, in winter season, grazing pasture was not suitable for lactating cows. The present finding is in consonance with other workers who observed the highest prevalence during the winter season [13,20]. Ketosis prevailed more commonly in small farms and was influenced by the seasonal stress factors [22]. In contrast, the present findings recorded higher prevalence rate amongst CHF cows during September and lowest in June in a population of 733 cows [15].

The prevalence scenario of ketosis at different monthly stages of lactation demonstrated that the intensity and dimension of the disease was more at the peak stage of lactation with gradual decrease of frequency at later stage [29]. The overall prevalence was also reflected in the same manner indicating the higher occurrence of ketosis during the 2-7 weeks after calving and the highest prevalence during the 1^st^ month of milk yield when the production potential remained at peak level causing severe economic loss to dairy handlers [27,30]. The first 2 weeks of lactation are the primary risk period for the development of subclinical ketosis, defined by serum BHBA concentration of 1.2 mmol/L or higher [31].

The present finding is in line with the earlier findings which reported that the prevalence of ketosis was the highest in 1^st^ month (i.e., from 0 to 30 days) followed by the 2^nd^ month (i.e., from 30 to 60 days) after calving [19,27]. The highest prevalence of 50.0% during 1^st^ and 2^nd^ month of lactation was recorded [18], and the prevalence of 42.8% during 1^st^ month followed by 28.6% during 2^nd^ month of lactation period [32] are in close proximity to the present findings. The highest prevalence has also been reported during the 1^st^ month of lactation which was in line with the present findings [2,27,28,33-36].

## Conclusions

The current field study has indicated a high prevalence of ketosis in dairy cows of small and marginal farmers, as these animals are unable to withstand the strains arising from the high nutritional demands generated by the production of milk resulting in development of hypoglycemia, and excess ketone bodies in blood giving rise to ketosis during the 1^st^ month of milk production at peak level. Ketotic milk cows exhibited anorexia, drastic reduction in milk yield, rapid loss of body condition, ketotic smell of milk with rapid breathing, etc. Farmers of Eastern India were well advised to provide good quality nutrition to reduce the possibility of cows in transition developing subclinical ketosis. In this study, the authors have given great attention for diagnosis, management, and control of ketosis during risk stage to prevent economic loss of the dairy farmers.

## Authors’ Contribution

SB and DCN designed the study, collected the sample and analyzed them. SB, DCN, and KKS provided technical guidance and participated in scientific investigations, data analysis, discussion, and drafted the manuscript. All authors have read and approved the final manuscript.
